# Reminder duration determines threat memory modification in humans

**DOI:** 10.1038/s41598-018-27252-0

**Published:** 2018-06-11

**Authors:** Jingchu Hu, Wenqing Wang, Philipp Homan, Penggui Wang, Xifu Zheng, Daniela Schiller

**Affiliations:** 1 School of Psychology and Center for Studies of Psychological Application, South China Normal University, Guangzhou, China; 2 School of Life Sciences, South China Normal University, Guangzhou, China; 3 Center for Psychiatric Neuroscience, The Feinstein Institute for Medical Research, Zucker School of Medicine at Northwell/Hofstra, Hempstead, NY USA; 4 Department of Psychiatry, Department of Neuroscience, and Friedman Brain Institute, Icahn School of Medicine at Mount Sinai, New York, NY USA

## Abstract

Memory reminders can return a memory into an unstable state such that it will decay unless actively restabilized into long-term memory through reconsolidation. Exposure to a memory reminder, however, does not always lead to destabilization. The ‘trace dominance’ principle posits that the extent of exposure to memory reminders governs memory susceptibility to disruption. Here, we provide a first systematic investigation of reminder duration effects on threat memory modification in humans. Reminder duration was parametrically varied across 155 participants in a three-day protocol. We found that short reminders (1 s and 4 s) made the memory prone to interference from post-retrieval extinction, suggesting that the memory had been updated. In contrast, no reminder or long reminders (30 s and 3 min) made the memory resistant to such interference, and robustly return. Reminder duration therefore influences memory stability and may be a critical determinant of therapeutic efficacy.

## Introduction

Upon retrieval, a memory may return to an unstable state and become amenable to modification. Memory reminders destabilize the memory such that it will decay unless actively restabilized into long-term memory. Reconsolidation refers to the molecular and cellular processes that returns the memory to a stable state^1,2^. Pharmacological manipulations of reconsolidation result in memory enhancement or impairment, and new information can get incorporated during reconsolidation into an updated memory^2–4^. Mere exposure to memory reminders, however, does not necessarily imply that the memory has entered an unstable state and therefore does not guarantee reconsolidation. There are cases of retrieved memories that are resistant to change^5–8^. Whether retrieval results in destabilization depends on a host of cellular and molecular mechanisms, which actively transfer the retrieved memory into an unstable neural representation. There appears to be a requirement for protein degradation, receptor-signaling activity, dendritic spine remodeling, gene regulation and epigenetic mechanism^7,9–20^.

At the behavioral level, the ‘trace dominance’ principle posits that the extent of re-exposure to reminders determines the dominance of the memory, and hence, its stability. This principle emerged as a theoretical framework for interpreting empirical data of associative memory modification^21^. In associative threat learning^22^, a conditioned stimulus (CS) is initially benign until paired with an aversive outcome (US), after which the presentation of the CS alone elicits defensive responses. Multiple presentations of the CS without the US will eventually induce extinction memory, which diminishes defensive reactions to the CS.

Using threat conditioning in the Medaka fish, Eisenberg and colleagues^21^ found that administering an amnesic agent (protein synthesis inhibitor) following a single reminder abolished the expression of threat memory, whereas administration after multiple retrievals (i.e., extinction) induced the opposite effect resulting in high expression of threat memory. Similar results have been observed in other species, including crab^23^, mice^24^, and rats^25,26^. According to the trace dominance interpretation, a single CS reminder makes the threat memory dominant and thus susceptible to interference. With multiple CS exposures, the extinction trace becomes dominant and hence vulnerable to disruption. This means that trace dominance and memory stability have an inverse relationship: reconsolidation interference will be effective following brief, but not extensive, reminder durations^27^.

Extinction learning in itself, as well as other behavioral protocols such as counterconditioning or new learning, can interfere with reconsolidation if timed to the post-retrieval period^3,4,28–30^. Extinction after a single CS reminder has been shown to update memories in various paradigms and across species^4,31^. Similar to pharmacological blockade of reconsolidation, memory modification via post-retrieval extinction also depends on initial memory destabilization and obeys the trace dominance principle in rodents^32,33^.

The critical conditions under which post-retrieval memory modification occurs are a topic of intense interest for clinical applications^4,34,35^. Based on pre-clinical studies, successful destabilization is a prerequisite for modification of maladaptive emotional memories via reconsolidation in a therapeutic setting. It is currently unknown, however, how reminder duration influences post-retrieval memory modification of threat memories in humans. We therefore set out to examine the trace dominance principle in 155 healthy participants using threat conditioning. Post-retrieval extinction served as the reconsolidation interference treatment following various reminder durations.

**Figure 1:**
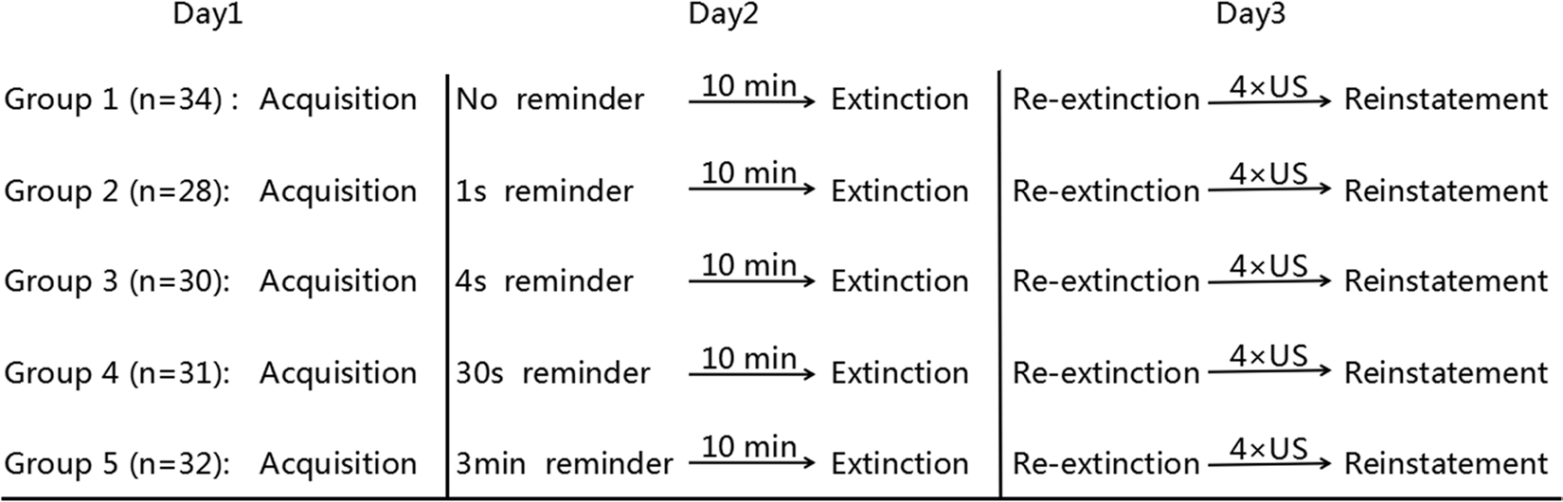
Schematic depiction of the experimental design.

The experiment was conducted over three days (Fig. 1). On day 1, the participants underwent threat conditioning, in which a 4 second visual stimulus (e.g., blue square) was paired with an electric shock on 38% of trials, whereas another visual stimuli (e.g., yellow square) was never paired with the shock. On day 2, the participants were randomly assigned into 5 experimental groups that differed in the reminder duration: the CS was presented for 0, 1, 4, 30 or 180 seconds. Approximately 10 minutes after the reminder session, all participants underwent extinction learning, which served here as the post-retrieval updating manipulation. On day 3, we examined the recovery of threat memory using re-extinction immediately followed by a reinstatement session. Skin conductance response (SCR) indexed conditioned defensive reactions. We examined the relationship between reminder duration and post-retrieval memory updating. Based on the trace dominance principle, we expected threat memory to recover following standard extinction (0 s reminder), but show a positive relationship with reminder duration such that longer durations will induce greater memory recovery.

## Results

### Day 1 - Conditioned threat acquisition

**Figure 2:**
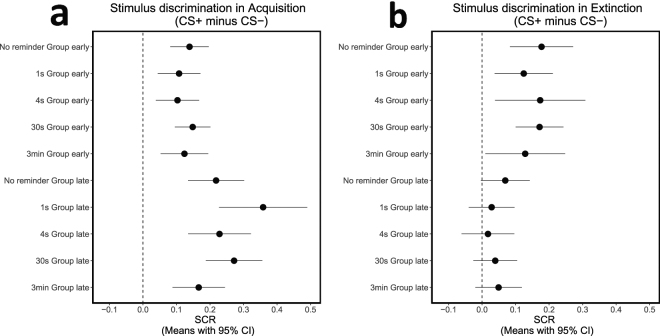
Stimulus discrimination in acquisition and extinction. Means with 95% confidence intervals in each group in the early and late phases of acquisition (**a**) and extinction (**b**). Confidence intervals that do not cross the vertical dashed line at zero indicate that the corresponding contrast is different from zero and thus statistically significant. The results show successful and similar acquisition and extinction in all groups.

To assure equivalent and significant acquisition across the randomly assigned groups (Fig. 2a), we conducted ANOVA with factors of group (5 reminder conditions) x phase (first and second halves of the acquisition session) x stimulus (CS+, CS−). There was no evidence for group differences as indicated by the non-significant main effect of group (*F*_4,150_ = 0.25, *P* = 0.907), group x stimulus interaction (*F*_4,150_ = 1.39, *P* = 0.242), group x phase interaction (*F*_4,150_ = 1.45, *P* = 0.221), or group x stimulus x phase interaction (*F*_4,150_ = 1.84, *P* = 0.125). SCR responses were higher to the CS+ compared to the CS− and this differential responding increased from early to late phase as indicated by a main effect of phase (*F*_1,150_ = 23.62, *P* < 0.001) and stimulus (*F*_1,150_ = 200.89, *P* < 0.001), and also a phase x stimulus interaction (*F*_1,150_ = 24.05, *P* < 0.001).

Follow-up *t-*tests confirmed significantly higher responding to the CS+ vs. CS− in all groups during all phases (early phase: no-reminder, *t*_33_ = 4.74, *P* < 0.001; 1 s reminder, *t*_27_ = 3.31, *P* = 0.003; 4 s reminder, *t*_29_ = 3.12, *P* = 0.004; 30 s reminder, *t*_30_ = 5.47, *P* < 0.001; 3 min reminder, *t*_31_ = 3.41, *P* = 0.002; late phase: no-reminder, *t*_33_ = 5.10, *P* < 0.001; 1 s reminder, *t*_27_ = 5.34, *P* < 0.001; 4 s reminder, *t*_29_ = 4.78, *P* < 0.001; 30 s reminder, *t*_30_ = 6.33, *P* < 0.001; 3 min reminder, *t*_31_ = 4.17, *P* < 0.001). These results confirm sufficient and similar acquisition across all groups.

### Day 2 - Conditioned threat extinction

To assure equivalent extinction across the groups (Fig. 2b), we conducted ANOVA with factors of group (5 reminder conditions) x phase (first and second halves of the extinction session) x stimulus (CS+, CS−). There was no evidence for group differences as indicated by the non-significant main effect of group (*F*_4,150_ = 0.15, *P* = 0.964), group x stimulus interaction (*F*_4,150_ = 0.25, *P* = 0.912), group x phase interaction (*F*_4,150_ = 2.27, *P* = 0.064), or group x stimulus x phase interaction (*F*_4,150_ = 0.29, *P* = 0.885). SCR responses were higher to the CS+ vs CS− and this differential responding decreased from early to late phase, as indicated by a main effect of phase (*F*_1,150_ = 227.18, *P* < 0.001) and stimulus (*F*_1,150_ = 36.96, *P* < 0.001), and also a phase × stimulus interaction (*F*_1,150_ = 20.96, *P* < 0.001).

Follow-up *t-*tests confirmed significantly higher responding to the CS+ vs. CS− in the first half of extinction in all groups (no-reminder, *t*_33_ = 3.69, *P* < 0.001; 1 s reminder, *t*_27_ = 2.81, *P* = 0.009; 4 s reminder, *t*_29_ = 2.52, *P* = 0.017; 30 s reminder, *t*_30_ = 4.71, *P* < 0.001; 3 min reminder, *t*_31_ = 2.12, *P* = 0.042; Fig. 2b), but no significant difference in any of the groups by the late phase of extinction (no-reminder, *t*_33_ = 1.86, *P* = 0.071; 1 s reminder, *t*_27_ = 0.82, *P* = 0.420; 4 s reminder, *t*_29_ = 0.44, *P* = 0.665; 30 s reminder, *t*_30_ = 1.18, *P* = 0.248; 3 min reminder, *t*_31_ = 1.39, *P* = 0.173), or by the last trial of extinction (no-reminder, *t*_33_ = 0.93, *P* = 0.360; 1 s reminder, *t*_27_ = 0.38, *P* = 0.705; 4 s reminder, *t*_29_ = 0.11, *P* = 0.911; 30 s reminder, *t*_30_ = 0.36, *P* = 0.721; 3 min reminder, *t*_31_ = 0.21, *P* = 0.838). These results confirm sufficient and similar extinction across all groups.

### Reminder duration influences post-retrieval memory modification

**Figure 3:**
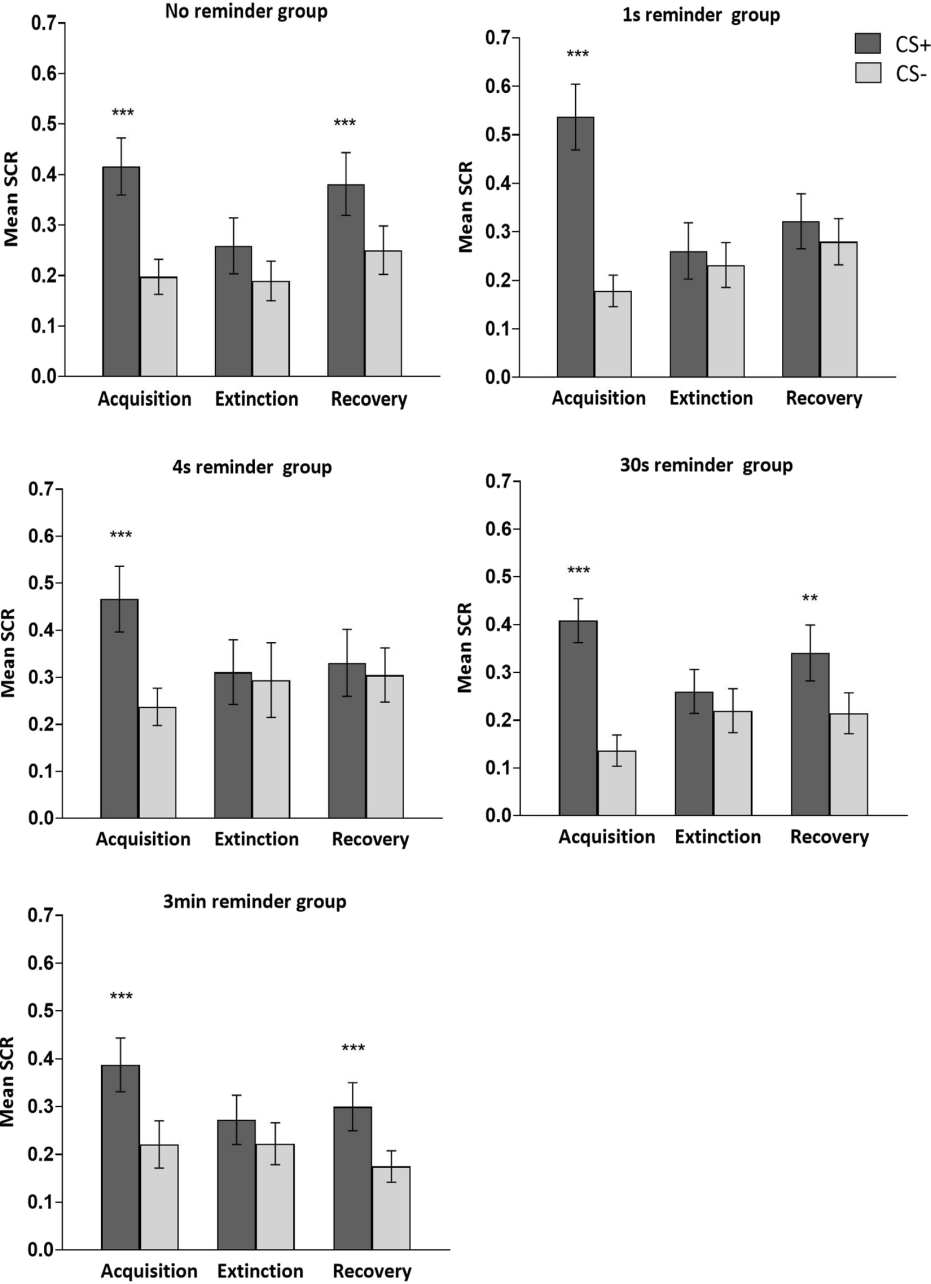
Short but not long reminder duration prevents memory recovery. Mean SCR (CS+ and CS−) during acquisition (late phase), extinction (late phase) and re-extinction (early phase) of the 5 experimental groups with different reminder durations. A significant 3-way interaction of group x stage x stimulus and follow-up *t*-tests confirmed memory recovery in the no-reminder, 30 s, and 3 min reminder groups, but not in the 1 s and 4 s reminder groups. **P < 0.01; ***P < 0.001. Error bars represent standard errors.

To assess the overall post-retrieval memory modification effect as a function of reminder duration (Fig. 3), we examined responses to the stimuli during threat learning (day 1, late acquisition) relative to extinction (day 2, late extinction) and recovery (day 3, early re-extinction). We thus conducted a 3-way ANOVA with factors of group (5 reminder durations), stage (acquisition, extinction, recovery) and stimulus (CS+, CS−), which yielded a significant three-way interaction (*F*_8,300_ = 2.02, *P* = 0.044).

Follow up *t* tests indicated that threat memory recovery was evident only in the no-reminder, 30 s reminder, and 3 min reminder groups (no-reminder, *t*_33_ = 3.91, *P* < 0.001; 30 s reminder, *t*_30_ = 3.18, *P* = 0.003; 3 min reminder, *t*_31_ = 3.92, *P* < 0.001), but not in the 1 s reminder or 4 s reminder groups (1 s reminder, *t*_27_ = 1.45, *P* = 0.158; 4 s reminder, *t*_29_ = 0.66, *P* = 0.516). These results indicate that reminder duration that was shorter or similar to the learned CS duration destabilized the memory, allowing updating with extinction, whereas longer duration rendered the memory resistant to modification.

### Memory expression relative to initial learning

**Figure 4:**
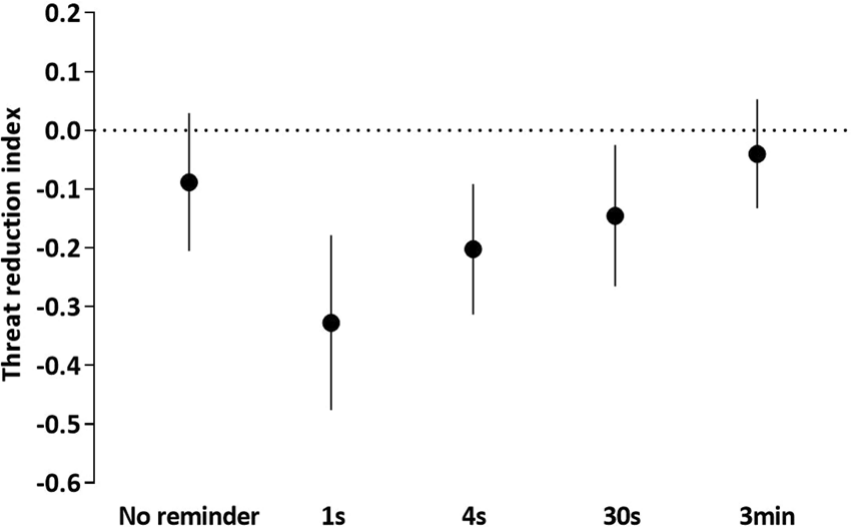
Short but not long reminder duration reduced stimulus discrimination from Day 1 to Day 3. Means with 95% confidence intervals in each group. Threat reduction index is the change in stimulus discrimination (CS+ minus CS−) from late acquisition to early re-extinction (Day 1 subtracted from Day 3). Confidence intervals that do not cross the vertical dashed line at zero indicate that the corresponding contrast is different from zero and thus statistically significant. The results show that on Day 3, the no-reminder and 3 min reminder groups expressed about the same level of threat discrimination acquired on Day 1 (threat reduction index around zero), whereas the 1 s, 4 s and 30 s reminder duration groups showed a significant reduction (negative threat reduction index) from acquisition to re-extinction. Note that in the 30 s reminder group (but not in the 1 s or 4 s reminder groups) stimulus discrimination remained significant despite this reduction.

A meaningful change in memory may be exhibited not only by lack of stimulus discrimination on Day 3, but also by a significant reduction in discrimination (CS+ minus CS−) relative to its initial acquisition (Fig. 4). To assess the degree of recovery on day 3 (early phase) relative to the degree of learning on day 1 (late phase), we conducted two-way ANOVA with factors of group (5 reminder durations) and stage (acquisition, recovery). This analysis revealed a significant interaction of group x stage (*F*_4,300_ = 3.42, *P* = 0.009). Follow-up *t*-tests confirmed that the no-reminder and 3 min group maintained stimulus discrimination to a similar degree between Day 1 and Day 3 (no-reminder, *t*_33_ = 1.53, *P* = 0.136; 3 min reminder, *t*_31_ = 0.88, *P* = 0.384), whereas the 1 s, 4 s, and 30 s reminder groups showed a significant reduction in stimulus discrimination from acquisition to recovery (1 s reminder, *t*_27_ = 4.52, *P* < 0.001; 4 s reminder, *t*_29_ = 3.73, *P* < 0.001; 30 s reminder, *t*_30_ = 2.47, *P* = 0.019). As mentioned above, however, the 30 s reminder group continued to show significant discrimination during Day 3 despite this reduction, whereas discrimination was lost in the 1 s and 4 s reminder groups.

### Persistence of post-retrieval memory modification

**Figure 5:**
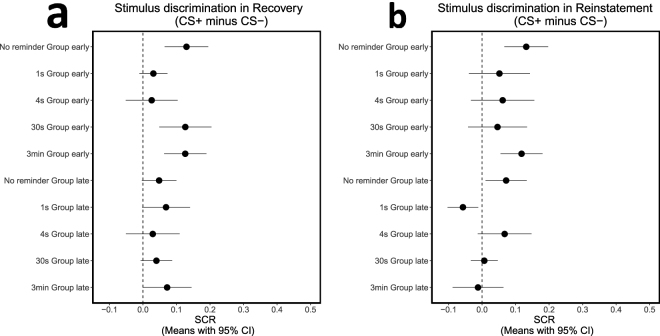
The effects of reminder duration persist through re-extinction and reinstatement. Means with 95% confidence intervals in each group in the early and late phases of re-extinction (recovery) and reinstatement. Confidence intervals that do not cross the vertical dashed line at zero indicate that the corresponding contrast is different from zero and thus statistically significant. The results show memory recovery (significant stimulus discrimination in early re-extinction) in the no-reminder, 30 s and 3 min reminder groups, but not in the 1 s and 4 s reminder groups. Stimulus discrimination was then extinguished in all groups (late re-extinction) and recovered only in the no-reminder and 3 min reminder groups (early reinstatement).

Following the early re-extinction session of Day 3, the participants continued the extinction session (late extinction) and then were exposed to 4 unsignaled shocks followed by another re-extinction session (reinstatement). A detailed analysis of these sessions (Fig. 5) revealed that those groups that did not show recovery in early re-extinction (1 s and 4 s) continued to exhibit lack of stimulus discrimination through late re-extinction (Fig. 5a; late phase: 1 s reminder, *t*_27_ = 1.88, *P* = 0.071; 4 s reminder, *t*_29_ = 0.71, *P* = 0.481) and reinstatement (Fig. 5b; early phase: 1 s reminder, *t*_27_ = 1.12, *P* = 0.274; 4 s reminder, *t*_29_ = 1.27, *P* = 0.213). Of the three other groups that showed memory recovery in early re-extinction (no reminder, 30 s reminder, and 3 min reminder), the no-reminder and 3 min reminder groups extinguished defensive responding by late re-extinction (Fig. 5a; late phase: no-reminder, *t*_33_ = 1.83, *P* = 0.077; 3 min reminder, *t*_31_ = 1.94, *P* = 0.062) but proceeded to show significant reinstatement following re-extinction (Fig. 5b; early phase: no-reminder, *t*_33_ = 3.96, *P* < 0.001; 3 min reminder, *t*_31_ = 3.69, *P* < 0.001). These results support the persistence of reminder duration effects on post-retrieval memory modification.

### Memory reminders diminish the correlation between extinction and recovery

**Figure 6:**
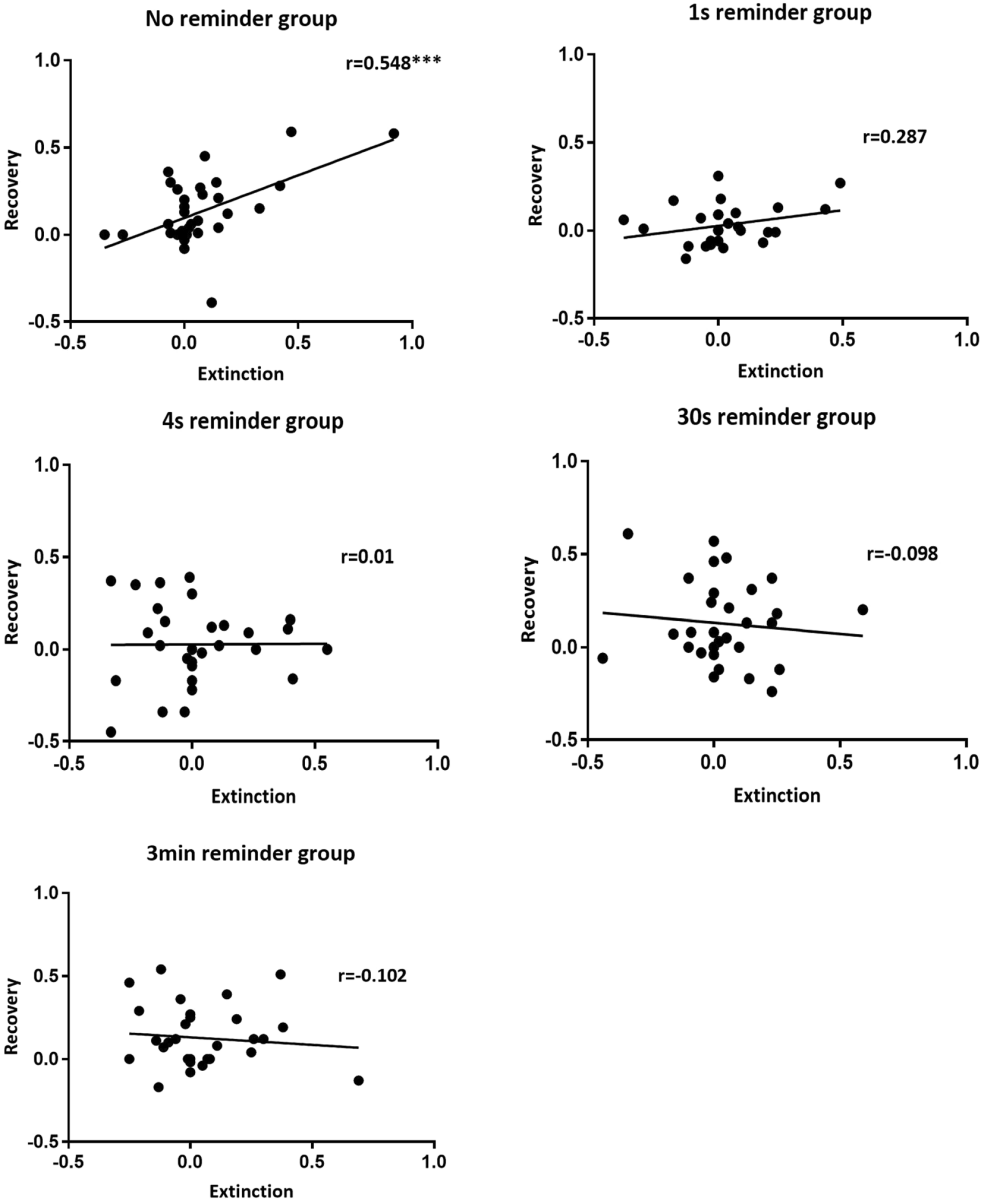
Memory reminders diminish the correlation between extinction and recovery. To examine the effects of reminder duration on the relationship between extinction learning the memory recovery we assessed the correlation between stimulus discrimination in Day 3 (early re-extinction) and stimulus discrimination in Day 2 (late extinction) in each group. Only the no reminder group showed a significant positive correlation between two parameters. There was no correlation in any of the groups with memory reminder. ***P < 0.001.

Finally, we assessed individual differences in extinction and recovery and whether the reminder influences the relationship between these two stages (Fig. 6). As demonstrated in rodents^26^, we would expect both parameters to correlate in standard extinction (i.e., no reminder), as participants with low differential SCR at the end of extinction should exhibit low discrimination in recovery. If retrieval-extinction impairs reconsolidation, however, we would expect an alteration of that correlation.

Consistent with these predictions and the rodent data^26^, we found a significant positive correlation (Pearson’s *r* = 0.548, *P* < 0.001) between extinction and recovery in the no-reminder group (the correlation remained significant also after removing a potential outlier, *r* = 0.399, *P* = 0.022), but not for any of the reminder groups (1 s reminder group, *r* = 0.287, *P* = 0.139; 4 s reminder group, *r* = 0.01, *P* = 0.958; 30 s reminder group, *r* = −0.098, *P* = 0.598; 3 min reminder group, *r* = −0.102, *P* = 0.580).

**Figure 7:**
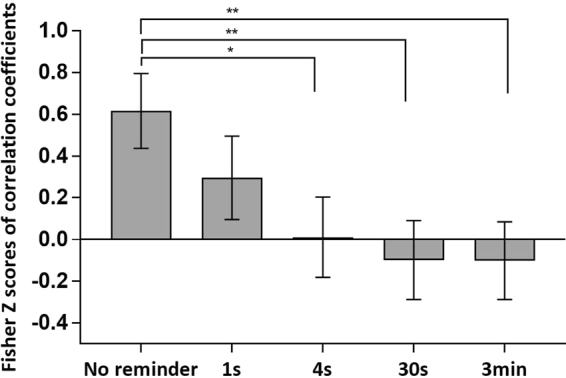
Memory reminders change the relationship between extinction learning and memory expression. Correlation coefficients of the experimental groups were compared by using Fisher *r*-to-*z* transformation. Significant differences were found between the no-reminder group and the 4 s, 30 s and 3 min reminder groups, but not for the 1 s reminder group. *P < 0.05; **P < 0.01. Error bars represent standard errors.

Comparing the groups’ correlation coefficients using Fisher *r*-to-*z* transformation (Fig. 7) indicated a significant difference between the no-reminder group and the 4 s (*z* = 2.3, *P* = 0.0214), 30 s (*z* = 2.74, *P* = 0.0061), and 3 min (*z* = 2.78, *P* = 0.0054) reminder groups; the difference with the 1 s reminder group did not reach significance (*z* = 1.19, *P* = 0.234). The results show that memory reminders change the relationship between extinction learning and memory expression, suggesting a qualitative change in memory representation.

## Discussion

We report here that short reminder durations, rather than long, were more effective in preventing the return of threat memory. Akin to having no reminder at all, long reminders produced a robust return of the defensive response. This pattern of results aligns with the dominant trace principle: brief reminder duration makes the threat memory dominant whereas extensive durations make the extinction trace dominate. The inverse relationship between trace dominance and memory stability predicts that short but not long reminders will render the memory unstable and prone to reconsolidation interference. We now demonstrate this relationship in human threat conditioning memory, adding to evidence accumulated in other species^21,23–26,36^.

In addition to reminder duration, previous studies delineated other boundary conditions on post-retrieval memory modification^37^. These conditions include an element of surprise in the reminder (i.e., prediction error), such as a temporal mismatch between the CS and US^38–40^; the age and strength of the memory^24,41–43^ and cue specificity^44,45^.

To account for all of these conditions, Gershman *et al*.^27^ developed the latent cause theory of conditioning, offering a unifying normative account of post-retrieval memory modification. At the heart of this theory is the idea that when encountering surprising events, the brain attempts to infer what caused them. Retrieving a memory pushes the brain toward inferring that an old cause is once again active, which means that the memory becomes eligible for updating. If the brain otherwise infers a new cause for the surprising event, a new memory will be formed.

The reminder duration effect features a central component of the theory: in absence of strong evidence to the contrary, the brain preferentially assigns new observations to causes previously inferred. Thus, limited exposure favors assignment of the reminder to the initial threat learning cause. With more extensive exposure to the reminder, prediction errors accumulate, nudging the brain to infer a new latent cause (e.g., ‘extinction’ or ‘safe’ cause). This is also seen in our results: short reminders (1 s and 4 s) made the memory prone to interference from post-retrieval extinction, suggesting that the memory had been updated and therefore did not return. In contrast, no reminder or long reminders (30 s and 3 min) made the memory resistant to such interference, and it thus robustly returned.

When examining individual differences in extinction and recovery, we found that memory reminders modified the relationship between extinction learning and memory expression: the correlation coefficients in participants that had reminders scaled negatively with reminder duration. Thus, the positive relationship between extinction learning and recovery, exhibited by the no-reminder group, became more negative with longer reminder durations. These findings suggest that despite the phenotypic similarity between no reminder and long reminders – as threat memory recovers following extinction in both cases – the nature of memory representation might be different. Borrowing from the latent cause model, it is possible that re-extinction following standard extinction triggers the previous ‘extinction’ cause. Long reminders, however, perhaps induce a new ‘safe’ cause that is different from the ‘extinction’ cause learned through extinction training in the absence of a memory reminder. This new ‘safe’ cause then incorporates extinction learning into a new memory representation. In other words, while both standard extinction and long reminders promote the formation of a new ‘safe’ memory, they do so via different mechanisms. These differences are manifested in the relationship between extinction and recovery: if re-extinction triggers the ‘extinction’ cause, the relationship between extinction and recovery will be preserved; if re-extinction triggers the ‘safe’ cause inferred during the long reminder, the relationship between extinction and recovery will diminish since recovery no-longer reflects what was learned in extinction but rather the formation of a ‘safe’ memory already at the reminder phase.

Demonstrating the trace dominance effect in human threat memory substantiates the relevancy of this principle to therapeutic settings. Exposure therapy, the gold-standard behavioral treatment for post-traumatic stress and anxiety disorders, is considered to reflect extinction learning mechanisms^46,47^. The structure of extinction training and its manifestation in the clinic may critically determine whether new learning or memory updating occurs^4,8^. This, in turn, can have an impact on the success of therapy, given that extinction learning per se appears difficult to remember following treatment^48,49^. This study suggests that clinicians may assess the efficacy of memory reactivation approaches that vary in degree, intensity and duration, as some techniques may foster emotional memory modification while others may hinder treatment.

## Materials and Methods

### Participants

All participants that completed the 3-day paradigm were included in the final analysis (n = 155; 48 men, 107 women; age range 18–22 years; mean age 19.78 ± 1.18. Sample size per group indicated in Fig. 1). Participants discontinued after day 1 (n = 17) if they had non-measurable SCR signal (<0.02 μS; n = 6) or showed an opposite pattern of responding to the stimuli (mean CS− > CS+ in late acquisition; n = 11). Participants were recruited from South China Normal University and were interviewed regarding their health and medical conditions. All participants were right-handed, had normal or corrected-to-normal vision, and free from any current or previous medical condition that would contraindicate participation (i.e., pregnancy; seizure disorder; cardiovascular disease). Participants were paid a small amount (¥50) for their participation in the experiment. The ethical committee of South China Normal University approved the study and informed consent was obtained from all participants. All experiments were performed in accordance with relevant guidelines and regulations.

### Apparatus and materials

#### Stimuli

We employed images of two squares with different colors (yellow, blue) that served as CS+ and CS−. The CS+ was paired with a mild shock (US) on a partial reinforcement schedule (38% of presentation), and the CS− was never paired with a shock. Assignment of colors to stimuli was counterbalanced across participants, and the images were identical in size and resolution. The US was a mild electric shock with a duration of 200 ms, delivered to the wrist of the non-preferred hand using a SD-9 Square Pulse Stimulator (made by Grass Technologies, an Atro-Med, Inc. Product Group) with a bead of conductance gel applied to each of the electrode heads. The subjects were asked to set the level of the shock themselves. In this procedure, a subjects was first given a very mild shock (10 V) which was gradually increased to a level the subject indicated as “uncomfortable, but not painful” with a maximum level of 60 V.

#### Skin conductance response

SCR was measured by the Spirit NeXus-10 BioTrace system. Two Ag/AgCI electrodes were attached to the tips of the second and third fingers of the participant’s opposite the hand of the shock electrode. The electrodes were connected to the GSR100 C module, which recorded SCR at 200 Hz. The level of SCR was assessed for each trial as the base-to-peak amplitude difference in skin conductance of the largest deflection (in microSiemens) in the 0.5–4.5 s latency window after stimulus onset. The minimal response criterion was 0.02 μS. Responses below this criterion were encoded as zero. The raw skin conductance scores were square root transformed to normalize the distributions, and scaled according to each subject’s mean square-root-transformed US response.

### Experimental procedure

The experiment was conducted over the course of three consecutive days, where the sessions were separated by approximately 24 h. During each day, participants sat behind a table with a 21-inch LCD monitor at a distance of 50 cm in a sound-attenuated and temprature-controlled room (25 °C). The monitor ran at a refresh rate of 60 Hz and had a resolution of 1024 × 768 pixels. The software package E-Prime 2.0 was used for stimuli presentation and BioTrace software was used for SCR data recording.

#### Day 1: Acquisition

Prior to the experiment, the participants signed the consent form and filled out an anxiety questionnaire (STAI; data not included). After attachment of the skin conductance and shock electrodes, the intensity of the US was determined. Participants were instructed to stay still and pay attention to the relationship between the squares and the shock. In the acquisition phase, the CS+ and CS− were presented 10 times each for 4s without the US; and additional 6 CS+ presentations co-terminated with the US. Stimulus order was pseudorandomized. Intertrial intervals (ITI) varied between 10 s, 11 s, and 12 s. At the conclusion of the day 1, participants were asked if they learned the relationship between CS and US (data not included).

#### Day 2: Reminder and Extinction

Participants were randomly assigned into five experimental groups: No reminder; 1 s reminder; 4 s reminder; 30 s reminder; and 3 min reminder. During the reminder phase, the CS+ was presented once (unreinforced) for the different durations. Participants were given a 10 min break and instructed to rest; the no-reminder group similarly stayed in the room for 10 min prior to the extinction session. Extinction followed and consisted of 11 nonreinforced presentations of the CS+ and CS− each in the no reminder group. For the other groups, there were 10 CS+ and 11 CS− presentations (since the CS− was not presented during the reactivation session, an extra presentation was added to the extinction session such that all CSs were presented an equal number of times during day 2). Stimulus order was pseudorandomized and counterbalanced across participants. Intertrial intervals varied between 10 s, 11 s, and 12 s.

Note that due to the reminder duration manipulation the total exposure time to the CS+ differs between the groups. This is unlikely to be a critical factor here, since both the no-reminder and 3 min reminder groups (shortest and longest total exposure times, respectively) showed equivalent levels of memory recovery.

#### Day 3: Re-extinction recovery test and Reinstatement test

During the recovery test, participants received 10 nonreinforced presentations of the CS+ and CS− each, in order to assess the extent to which conditioned defensive responses could be recovered. After 18 s, a reinstatement session commenced where participants were administered with 4 unsignalled shocks (ITI 10-12 s), and then presented with additional nonreinforced presentations of 10 CS+ and 10 CS−. Stimulus order was pseudorandomized and counterbalanced across participants. Intertrial intervals varied between 10 s, 11 s, and 12 s.

### Statistical analysis

Skin conductance response was analyzed using a mixed analysis of variance (ANOVA) for repeated measures with reminder duration as a between-subjects factor, and stage and stimulus as within-subjects factors. The specific ANOVAs are indicated where appropriate. The significance threshold was set at 0.05, two-tailed.
