# Author Correction: Reminder duration determines threat memory modification in humans

**DOI:** 10.1038/s41598-018-36665-w

**Published:** 2018-12-06

**Authors:** Jingchu Hu, Wenqing Wang, Philipp Homan, Penggui Wang, Xifu Zheng, Daniela Schiller

**Affiliations:** 1 School of Psychology and Center for Studies of Psychological Application, South China Normal University, Guangzhou, China; 2 School of Life Sciences, South China Normal University, Guangzhou, China; 3 Center for Psychiatric Neuroscience, The Feinstein Institute for Medical Research, Zucker School of Medicine at Northwell/Hofstra, Hempstead, NY USA; 4 Department of Psychiatry, Department of Neuroscience, and Friedman Brain Institute, Icahn School of Medicine at Mount Sinai, New York, NY USA

Correction to: *Scientific Reports* 10.1038/s41598-018-27252-0, published online 11 June 2018

This Article contains errors.

In the Methods section under subheading ‘Experimental procedure’; in the section titled ‘Day 2: Reminder and Extinction’,

“Extinction followed and consisted of 11 nonreinforced presentations of the CS+ and CS− each in the no reminder group.”

should read:

“Extinction followed and consisted of 10 nonreinforced presentations of the CS+ and 11 CS− presentations in the no reminder group (the last trial was a CS-, not included in analysis).”

“Stimulus order was pseudorandomized and counterbalanced across participants”

should read:

“One pseudorandomized stimulus order was used, where cue colors were counterbalanced across participants.”

In the section titled ‘Day 3: Re-extinction recovery test and Reinstatement test’,

“During the recovery test, participants received 10 nonreinforced presentations of the CS+ and CS− each, in order to assess the extent to which conditioned defensive responses could be recovered. After 18 s, a reinstatement session commenced where participants were administered with 4 unsignalled shocks (ITI 10-12 s), and then presented with additional nonreinforced presentations of 10 CS+ and 10 CS−. Stimulus order was pseudorandomized and counterbalanced across participants.”

should read:

“During the recovery test, participants received 10 nonreinforced presentations of the CS+ and 11 CS− presentations. After 18 s, a reinstatement session commenced where participants were administered with 4 unsignalled shocks (ITI 10-12 s), and then presented with additional nonreinforced presentations of 10 CS+ and 11 CS− presentations. One pseudorandomized stimulus order was used, where cue colors were counterbalanced across participants. The first trial was a CS-, not included in analysis.”

In addition, the Data Availability statement was omitted from the Additional Information section:

Data can be found at Open Science Framework: osf.io/frzv7

